# The value of mammography in women with focal breast complaints in addition to initial targeted ultrasound

**DOI:** 10.1007/s10549-020-05943-5

**Published:** 2020-09-30

**Authors:** L. Appelman, P. T. M. Appelman, C. C. N. Siebers, P. Bult, H. L. S. Go, M. Schlooz, R. M. Mann

**Affiliations:** 1 Department of Medical Imaging, Radboud University Medical Center, Nijmegen, The Netherlands; 2 Department of Radiology, St. Antonius Hospital, Leidsche Rijn, The Netherlands; 3 Department of Pathology, Radboud University Medical Center, Nijmegen, The Netherlands; 4 Department of Radiology, Noordwest Ziekenhuisgroep, Alkmaar, The Netherlands; 5 Department of Surgery, Radboud University Medical Center, Nijmegen, The Netherlands; 6 Department of Radiology, the Netherlands Cancer Institute, Amsterdam, The Netherlands; 7 Department of Radiology and Nuclear Medicine, Radboud University Medical Centre, Geert Grooteplein 10, Post 766, 6525 GA Nijmegen, The Netherlands

**Keywords:** Focal breast symptoms, Initial targeted ultrasound, Mammography

## Abstract

**Purpose:**

To determine the added value of mammography in women with focal breast complaints and the utility of initial targeted ultrasound in this setting.

**Methods:**

Women with symptomatic breast disease who were evaluated by breast imaging (mammography/digital breast tomosynthesis and ultrasound) between January 2016 and December 2016 in the Radboud University Medical Centre were included. We retrospectively collected the following data: date of birth, indication of imaging, visibility on mammography/ultrasound, whether biopsy was taken, additional findings, BI-RADS-classification, pathology and follow-up results.

**Results:**

A total of 494 women were included (mean age 46.5, range 30 to 93). In 49 women (9.9%), symptomatic breast cancer was diagnosed, all visible during targeted ultrasound. The negative predictive value of targeted ultrasound was very high (99.8%). Additional findings on mammography were significantly more often malignant when the symptomatic lesion was also malignant (3.8% vs 70%, *P* < 0.05). In only one patient with symptoms caused by a benign finding, an incidental malignancy was detected on mammography outside the area of complaint (detection rate 2.2/1000 examinations).

**Conclusions:**

The contribution of mammography for cancer detection in women with focal breast complaints is very low when targeted ultrasound is performed. Additional findings are most common in patients with symptomatic breast cancer. Our results suggest that initial targeted ultrasound is a more appropriate initial tool for the evaluation of focal breast complaints. Mammography could be performed on indication only.

## Introduction

In the Netherlands per 1000 women, 29.7 women with focal breast symptoms visit a general practitioner per year. The majority of visits is due to a palpable mass, but also skin changes, nipple changes, nipple excretion and focal pain are included. The highest incidence of focal breast complaints is found in women aged 25 to 44 years (48 per 1000) [1]. Although the exact fraction of these women that is referred to a breast clinic is unknown, roughly 70.000 women (1/3rd) with focal breast complaints present at a radiology department in the Netherlands annually.

For women over 30, the baseline examination includes mammography (and digital breast tomosynthesis in most hospitals) followed by targeted ultrasound at the spot of the focal breast complaint. Biopsy is performed when needed according to various national guidelines [2, 3]. The role of breast ultrasound has gradually increased in this evaluation due to advances in the technology [4] and improvement of interpretation of ultrasound findings by breast radiologists.

The Sydney Breast Imaging Accuracy Study suggests that ultrasound may be an appropriate initial imaging examination in young women (< 45 years) [5]. Lehman et al. [6] likewise concluded that targeted ultrasound has a high sensitivity (95.7%) and high negative predictive value (NPV) (99.9%) in women between 30 and 39 years, and should be the primary imaging modality of choice. They noted that the added value of mammography is very low [6, 7]. In a study by Leung et al. [7] it was concluded that there is no added value for repeating mammography when a negative mammography performed within 12 months is already present. It appears that the added value of mammography mostly lies in the determination of multifocality and/or the extent of disease in case of breast malignancy [8]. Furthermore, mammography may be helpful when the radiologist is uncertain about the ultrasound findings or in case of a discrepancy between the clinical assessment and the ultrasound.

Still, these studies only focused on relatively young women, and do therefore not inform on the effect of age for supplemental cancer detection with mammography. This retrospective study aims to assess the diagnostic accuracy of targeted ultrasound and the added value of mammography in all women over 30 years of age presenting with a focal breast complaint.

## Methods

We performed a retrospective cohort study to assess the value of mammography and targeted ultrasound in women presenting with focal breast complaints at our department.

### Patients

We searched the Picture Archiving and Communication System (PACS) for all patients who underwent a mammography and targeted ultrasound at the same day at our department between the 1st of January and 31st of December 2016.

**Fig. 1:**
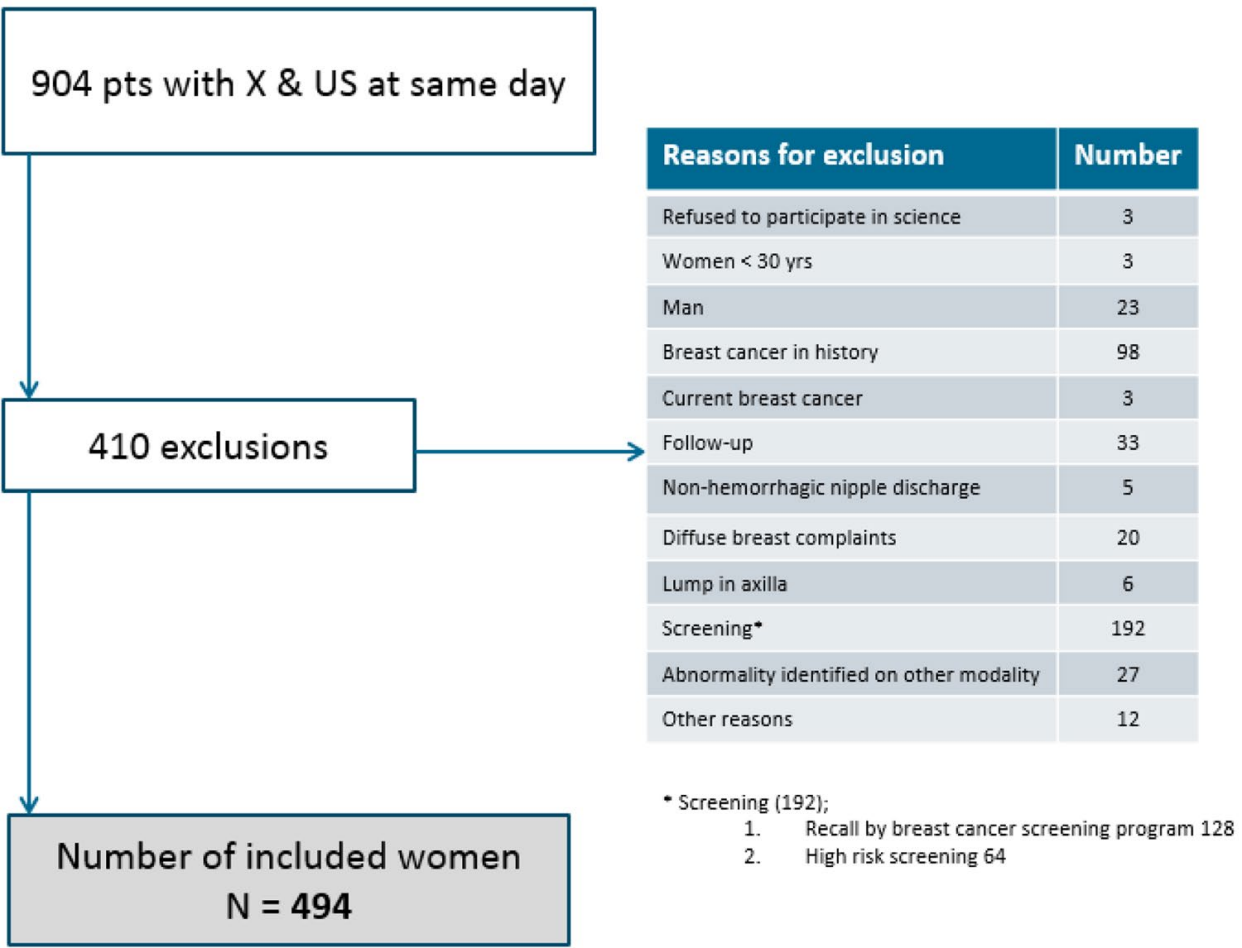
Exclusion criteria

This yielded 904 patients. 410 patients were subsequently excluded for various reasons as listed in Fig. 1. The final study cohort therefore consisted of 494 women.

### Imaging

All patients underwent regular bilateral mammography and/ or digital breast tomosynthesis consisting of a two-view (craniocaudal and mediolateral oblique) examination of both breasts using a Siemens MAMMOMAT Inspiration VB60 with Prime Technology (Siemens, Erlangen, Germany). All mammograms were automatically scored for breast density using Volpara software (version 3.4.1/1.5.5.1.) (Volpara solutions, Christchurch, New Zealand).

All targeted ultrasound examinations were performed by 1 of 5 dedicated breast radiologists with 3–26 years of experience using a SIEMENS Healthcare Acuson S2000 Ultrasound system with a preset mode for breast imaging.

### Data extraction

For all women we obtained the radiological assessment from the reports, including a full lexicon description of eventual findings according to the Breast Imaging Reporting and Data System (BI-RADS) Lexicon, and a BI-RADS score for both mammography and targeted ultrasound [9]. In case of incomplete or unclear data in the report, images were reviewed by a breast radiologist (L.A.) with 4 years of experience, if needed another experienced breast radiologist was consulted to obtain consensus.

For all patients the following items were scored: indication of the examination, mammographic density, presence of an abnormality at the focal complaint site on mammography and ultrasound, type of abnormality at the focal complaint site for mammography and ultrasound, morphologic characteristics on both imaging modalities, suspicion of malignancy regarding the focal complaint site scored by BI-RADS classification, additional findings on imaging (mammography and ultrasound separately), performance of biopsy for the focal complaint, performance of biopsy for additional findings, lesion histopathology.

Data was collected in Castor EDC, a cloud-based Electronic Data Capture system (Castor EDC, Amsterdam, NL).

### Data interpretation and ground truth

BI-RADS 1 and 2 were considered as a negative result and BI-RADS 3, 4 and 5 were considered as a positive result. Ground truth was obtained from the histopathological reports when a breast biopsy was performed. A small group of women (*n* = 3) underwent magnetic resonance imaging (MRI) of the breasts for further analysis. For all women at least 2 years of negative follow-up was used to confirm the absence of breast cancer. Follow-up was deemed negative in case women didn’t come back for the same complaint, the lesions were downgraded after a period of follow-up (downgraded to BI-RADS 2) and if women were referred again with the same focal breast complaint and still underwent biopsy that was negative for breast cancer. Follow-up was regarded positive when a woman developed breast cancer in the 2 years after inclusion, regardless of her previous focal breast complaint.

### Statistics

The primary outcome of this study was the frequency of unexpected malignant findings diagnosed by mammography alone. In these women the breast complaints are explained by normal or benign results according to the targeted ultrasound, but the mammography revealed a misinterpreted abnormality at the focal spot of complaint or revealed an abnormality in a non-symptomatic area. Sensitivity, specificity, negative and positive predictive value of both modalities combined and targeted ultrasound alone were calculated. We considered mammography to be beneficial in cases where it had a clinical consequence that was different from the recommendation of the ultrasound examination and led to the detection of a histopathological proven malignancy.

We compared cancer yield and frequency of false positive findings between imaging modalities using chi-square and McNemar tests. 95% confidence intervals (CI) were calculated. A *p*-value of < 0.05 was considered significant. The statistical analyses were performed in IBM SPSS Statistics Version 25.

## Results

**Fig. 2:**
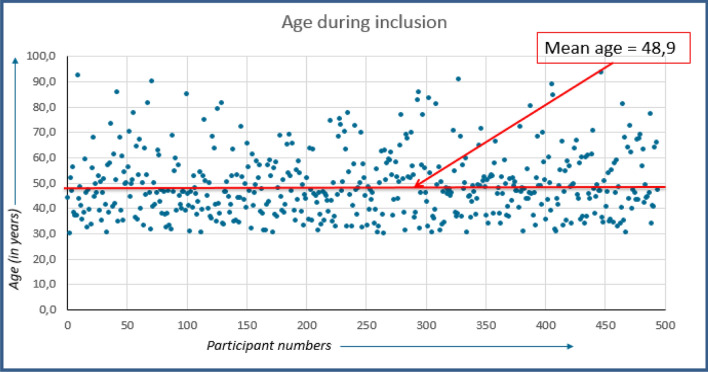
Age during inclusion

The median age of our population (*n* = 494) was 46.5 years (range 30.2–93.7 years) (Fig. 2).

The clinical indications for breast imaging were divided into five subgroups: focal lump (82.0%), focal skin or nipple retraction (7.3%), hemorrhagic nipple discharge (3.2%), focal pain or “different feeling” (6.9%) and nipple eczema (0.6%).

**Fig. 3:**
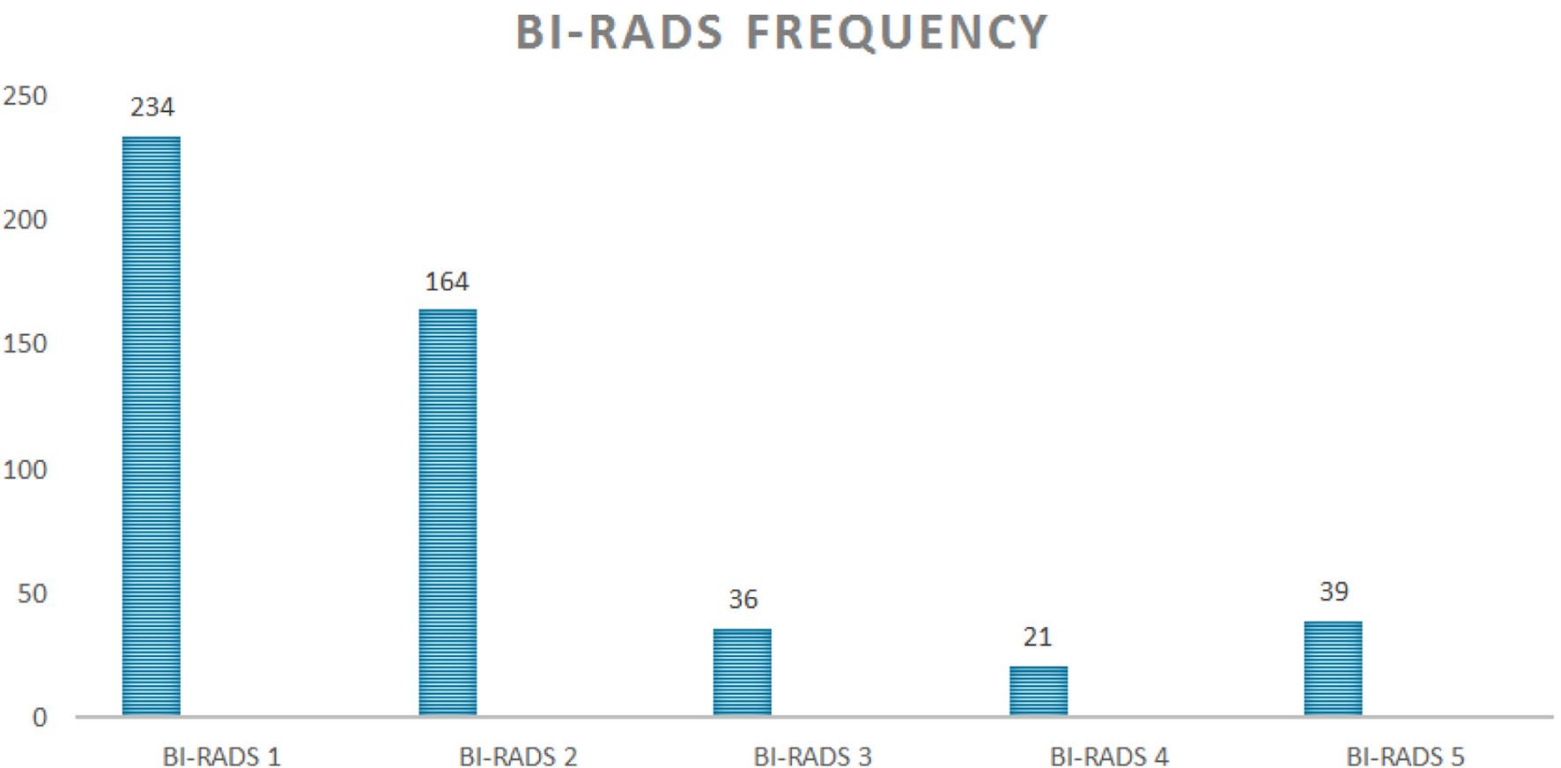
Frequency of BI-RADS scores given in the analysis of focal breast complaints

Most of the women (80.6%, average 47.5 years) had a negative or benign result after imaging. According to the reports of mammography and ultrasound, complaints were caused by normal fibroglandular tissue or a benign lesion at the focal spot, respectively classified as BI-RADS 1 (*n* = 234) and BI-RADS 2 (*n* = 164). 19.4% of cases was classified as BI-RADS 3, 4 or 5 (Fig. 3).

Combining both modalities, 260 (52.6%) women were diagnosed with a symptomatic lesion that correlated with the complaint. Targeted ultrasound showed 248 symptomatic abnormalities (varying from BI-RADS 2 to BI-RADS 5 lesions) whereas only 161 symptomatic abnormalities were visible on mammography (Table 1). In other words, in 99 cases (99/494: 20.0%) the (symptomatic) breast lesion was only visible on ultrasound and mammographically occult.

**Table 1:** Imaging of focal complaint

Imaging complaint	Normal	Abnormality	Size (mm)	BI-RADS 2	BI-RADS 3	BI-RADS 4	BI-RADS 5	Biopsies	Malignant
MG	333 (67.4%)	161 (32.6%)	7.2	82 (50.1%)	24 (14.9%)	17 (10.6%)	38 (23.6%)	79 (49.1%)	47 (29.2%)
US	246 (49.8%)	248 (50.2%)	8.6	164 (66.1%)	36 (14.5%)	21 (8.5%)	39 (15.7%)	83 (33.5%)	49 (19.8%)

164 of the 260 visualized lesions (63.1%) were unambiguously benign based upon diagnostic imaging (i.e. classified as BI-RADS 2).

There were 96 examinations (96/494:19.4%) classified as BI-RADS 3, 4 or 5, all visible on targeted ultrasound. A total of 91 ultrasound guided biopsies were performed in 86 women to evaluate abnormalities that correlated with the focal breast complaint(s). In five women two different symptomatic lesions were biopsied. The remainder consisted of eleven BI-RADS 3 lesions, which underwent short term follow-up, except for one woman. This specific young woman (30 years of age, presenting during lactation period) presented with a lump that was already getting smaller. According to the ultrasound imaging her complaint was probably caused by a galactocele (also getting smaller compared to ultrasound imaging in the previous year). She was recommended to return only when the mass would grow. There was one case which turned out to be more suspicious after a second ultrasound two months later, the BI-RADS classification was changed to BI-RADS 4a and it turned out to be malignant (lobular carcinoma) after biopsy and histopathological analysis. All other BI-RADS 3 lesions were subsequently downgraded to BI-RADS 1 or 2. The women (3) who underwent an MRI for further analysis of their breast complaints were eventually reassured. In one case non-mass enhancement with wash-out at the location of the complaint, MR guided biopsy was needed to exclude malignancy (pathology: adenosis). The other two MRIs showed no enhancement at the site of the complaint and were regarded as conclusive.

51 of 96 examinations (53.1%) were proven to be malignant (mean size: 22 mm on ultrasound, range 3 to 50). Characteristics of detected cancers and biopsied benign lesions are presented in the Appendix Table 2.

**Fig. 4:**
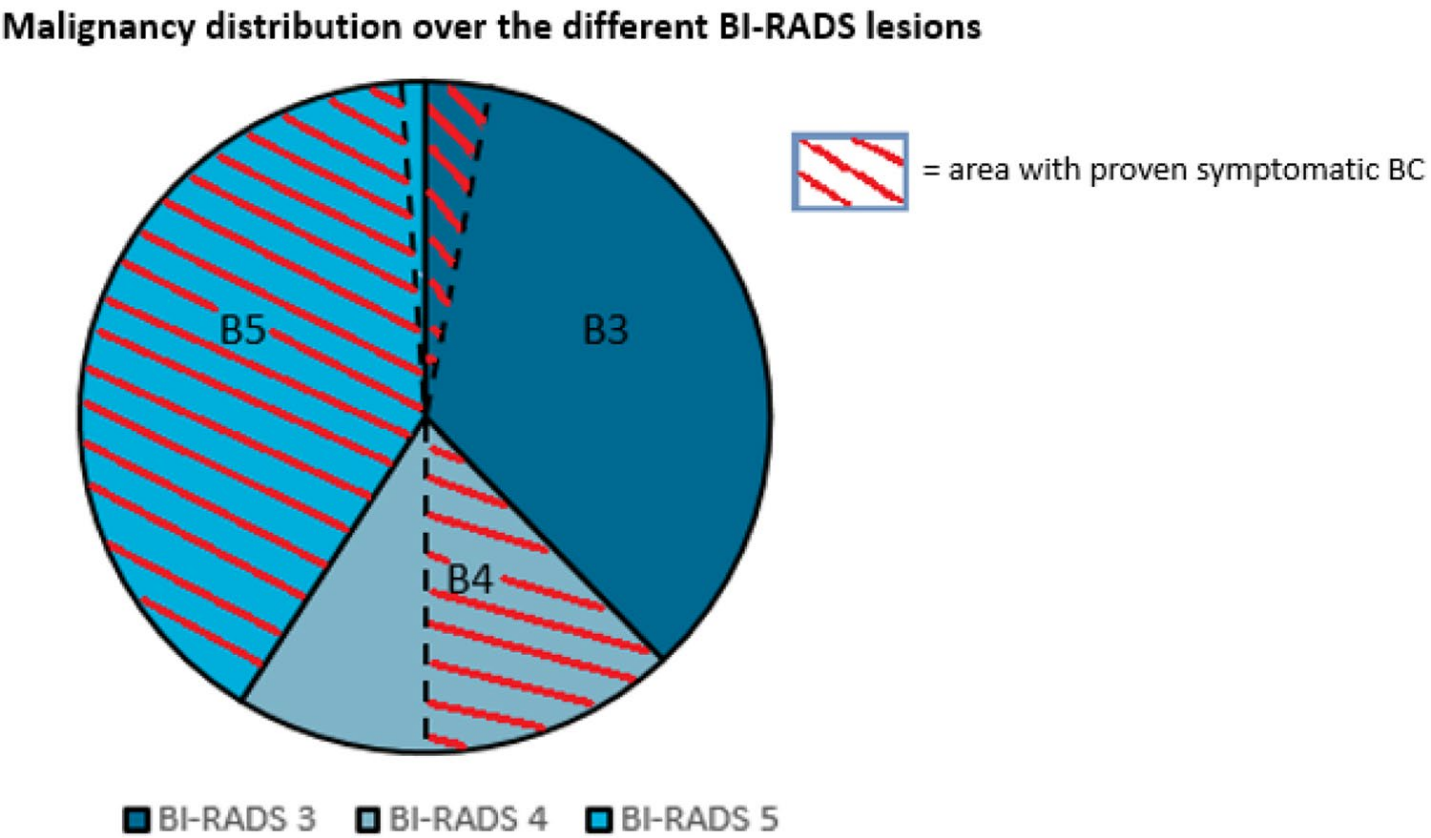
Malignancy at the focal complaint site, pathologically proven

Thirty-nine lesions were classified as BI-RADS 5, of which 38 turned out to be malignant (the last was a radial scar without signs of malignancy as proven by surgical excision) (PPV 97.4%; 38/39). Nine of 20 BI-RADS 4 lesions turned out to be malignant (PPV 45.0%), and 2 of 36 BI-RADS 3 lesions (PPV 5.6%) (Fig. 4). In total 49 symptomatic breast cancers were thus found during clinical imaging work-up of the focal complaint, excluding additional findings and malignancies detected during follow-up.

In summary, all symptomatic malignancies were visible during targeted ultrasound and the radiologists classified all malignant lesions as BI-RADS 3 or higher (i.e. local sensitivity was 100%). Two cancers were occult on mammography; hence, the local sensitivity of mammography was 95.9% (47/49).

There was no significant difference in cancer detection between the different indications. The presence of a lump was the main presented complaint (82.0%; 405/494). When biopsies were taken there was a 54.3% chance of malignancy (38/70). The relative risk of having breast cancer in case of a lump was 9.4% (38/405). In women with “focal skin/nipple retraction” (36/494) all performed biopsies yielded malignant results (100%; 10/10). This complaint resulted in the detection of malignancy in 27.8% (10/36). The relative risk of having breast cancer in case of “focal pain/ different feeling” was 3% (1/34). In our study we did not observe breast cancer in the group of women with indication “hemorrhagic nipple discharge” (15/494) and “nipple eczema” (3/494).

### Additional findings

In 294 cases (59.5%: 294/494) an additional finding was reported, defined as an abnormality on imaging that did not correlate with the focal breast complaint. 268 (90.9%: 267/294) lesions were classified as BI-RADS 2 and usually reflected benign calcifications or cysts away from the area of the focal complaint. The remaining 35 additional findings in 34 women were deemed suspicious [14 BI-RADS 3 (4.8%; 14/295), 17 BI-RADS 4 (5.8%:17/295) and 4 BI-RADS 5 (1.4%:4/295)]. Eleven suspicious additional findings were reported in women with cancer at the focal complaint, whereas 25 suspicious findings were reported in women with benign findings at the site of complaint.

**Fig. 5:**
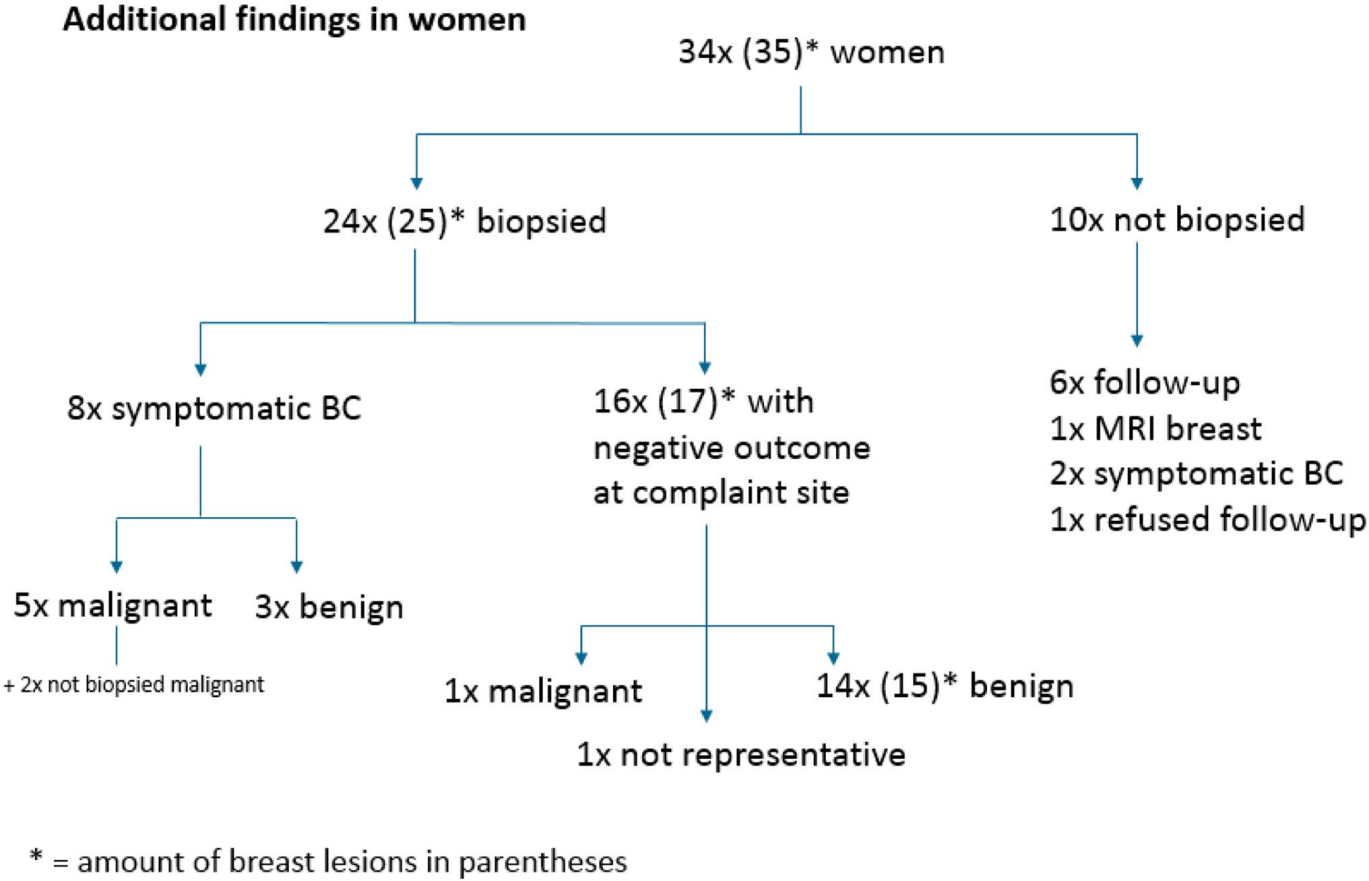
Additional findings

Twenty-three women underwent biopsy for an additional finding (8 with malignancy and 16 without malignancy at the focal complaint site), whereas supplemental imaging (MRI *n* = 1) and follow-up (*n* = 10) were used in the other women (all without malignancy at the focal complaint site). In two women with an additional lesion (a BI-RADS 4 and a BI-RADS 5 lesion) no additional biopsy was performed because malignancy was detected at the site of the complaint, and this finding would not alter management. However, the finding was shown to be malignant at subsequent histopathological evaluation of the surgical specimen in one patient (with an BI-RADS 5 lesion at the focal complaint site). The other patient who did not undergo additional biopsy was a 92-years old woman (with an BI-RADS 5 lesion at the focal complaint site) who didn’t undergo surgery because of the chosen palliative policy. All suspicious lesions that underwent (only) follow-up were eventually downgraded to BI-RADS 1 or BI-RADS 2, except for one woman with additional suspicious calcifications who refused further analysis or follow-up (Fig. 5). The additional finding (indistinct mass on ultrasound) that was evaluated with MRI showed normal breast tissue in this specific area, no malignancy was found.

Twenty-four women underwent biopsy for the additional lesions detected on mammography (8 lesions in women with cancer and 17 lesions in women without cancer at the focal complaint site). Eighteen biopsies yielded benign results. One biopsy of a cluster calcifications was not representative but was subsequently regarded as benign (classified as BI-RADS 2) after a follow-up period of 2 years and 9 months with mammography (Fig. 5).

Overall, additional findings were significantly more often malignant when the symptomatic lesion was also malignant (5/8) than when the symptomatic lesion was benign (1/17), *p* < 000.1. The negative predictive value of targeted ultrasound is very high (99.8%). The only woman (1/494; 0.2%) who was diagnosed with a malignancy while presenting with benign complaints, was referred for a focal skin retraction in her right breast, which turned out to be Mondors’ disease based on mammographic and targeted ultrasound evaluation. The mammography, however, showed a small area (6 mm) of clustered calcifications in the contralateral breast (left side). Stereotactic biopsy revealed ductal carcinoma in situ (DCIS) grade I–II. This detection had no clinical consequences (a wait-and-see policy was chosen), because she was already diagnosed with metastatic ovarian cancer. She eventually died within 1 year from that disease.

In women under 40 years of age (135/494) only two (mean age of 36.5 years) were diagnosed with breast cancer. Both presented with focal breast complaints at the site of the cancer. In this age category eight reports mentioned additional findings classified as BI-RADS 3 or more after the assessment of mammography and further analyses all showed benign outcome.

### Follow-up

Two patients developed breast cancer after initial benign findings (2/445) within 2 years from presentation. One 65-year-old woman originally presented with a lump in her left breast, which was biopsied and diagnosed as benign intraductal papilloma. She returned to our department 12 months later for re-evaluation because of growth of the lump. Repeat biopsies confirmed intraductal papilloma but now with the presence of a DCIS component, which led to subsequent surgical removal of the papilloma. The other patient (a 37-year-old woman) was initially diagnosed with a cyst (size 9 mm), explaining the focal complaints at 9 o’clock in her left breast. She returned ten months later with a small lump in the same breast, although this time at 3 o’clock. This new lump was seen as a small oval hyperdense mass, sharply demarcated on the mammography and in retrospect unchanged compared to the mammogram (Volpara density score C) obtained ten months before. The ultrasound showed a non-cystic suspicious lesion, which was biopsied and yielded invasive ductal carcinoma with DCIS (size 6 mm). Subsequent breast MRI revealed three small tumors in her left breast; one ductal carcinoma and two lobular carcinomas at histopathological analysis.

All additional findings (> BI-RADS 3) without biopsy were downgraded to BI-RADS 1 or 2 after follow-up.

## Discussion

In this retrospective study, we demonstrated that breast radiologists accurately evaluated focal breast complaints by targeted ultrasound only. Although previous studies are limited to a certain age category, our patient population consists of all women over 30 years of age. All symptomatic malignancies were visible during targeted ultrasound and were classified as “BI-RADS 3 or more” lesions. Even all lobular carcinomas (*n* = 7) were visible on targeted ultrasound in our study. In fact, the sensitivity of targeted ultrasound is higher compared to mammography in our study population. This is consistent with other studies [10, 11]. In total 35 additional suspicious findings were found by mammography alone, including 6 malignant lesions. Of these, one malignancy (DCIS) was detected on mammography while the complaint was explained by a benign lesion. All other additional malignant lesions were found in cases with (already) symptomatic breast cancer.

We observed only a small difference between the final diagnoses causing the focal complaints by targeted ultrasound and mammography in terms of cancer versus non-cancer (100 vs. 95.9%). Noteworthy is, however, that around 35% of the symptomatic (predominantly benign) abnormalities were occult on mammography. This may be explained by the high amount of dense breasts (70.3% breast density Volpara C or D) in our study population, which might be due to the relatively younger age of women presenting with focal breast complaints than is typical for screening populations.

A much bigger difference between the two modalities was observed in the detection of additional findings away from the focal complaint site. Targeted ultrasound led to detection of supplemental abnormalities in 28.7% (142/494) of cases (mainly cysts in the surrounding breast tissue), whereas mammography detected additional findings in 55.5% (274/494). However, most additional findings were clearly benign (e.g. calcifications with a benign aspect) and did not change clinical management.

The perceived added value of mammography is the detection of non-symptomatic malignant lesions in women presenting with focal complaints elsewhere. However, according to our analysis the detection rate of additional cancer in women without cancer at the spot of the focal complaint is very low (1/494), even when compared to our national breast cancer screening program (approximately 6/1000 (National Evaluation team for Breast Cancer screening, 2014)). This might be caused by the fact that the women presenting with focal breast complaints were on average younger (mean 48.9 years of age) than women in the screening population (50–75 years of age), which reduces both the frequency of breast cancer and the sensitivity of mammography. Moreover, our lower rate of additional cancer than expected, could be due to the relatively high participation in the National screening program, which can lead to a slight selection bias with regard to the group of women who are eligible for screening.. About one third of the study population (167 women) would be eligible for screening according to the national guidelines (women aged ≥ 50 and ≤ 75). We do not know exactly which women participated in the screening program. However, our low detection rate of additional cancer equals the results of a study in Ireland [12]. They reported detection of 2,1 per 1000 additional cancers, although their study was limited to a younger age category (35–39).

In women with breast cancer at the spot of the focal complaint, mammography detected additional malignant lesions (multifocal disease) in a fair number of patients (5/8; 62.5%). Mammography in this setting should therefore be regarded as a staging examination that may better show the extent of disease than targeted ultrasound, and may show contralateral breast cancer in women at increased risk. Hence, there is obvious value in the performance of mammography in women with focal complaints diagnosed as cancer. However, other studies have shown that for staging purposes, mammography may not be the best tool, and for example breast MRI has a much better concordance with histopathological examination [13].

The performance of mammography in women with focal abnormalities does lead to a substantial number of false positive findings. In our study 28 women (28/445; 6.3%) underwent further analyses because of the detection of an additional finding on mammogram, with afterwards benign outcome (such as complex cyst, pseudoangiomatous stromal hyperplasia, intraductal papilloma, fibroadenoma and adenosis). Logically, the biopsy rate increases with age with regard to the additional findings. The biopsy rate for additional findings in our study population with a benign outcome of the complaint is higher than to the biopsy rate of the Ireland study (3.8% vs. 2.8%) [12], likely due to the higher average age in our population. Apart from the (in retrospective unnecessary) benign biopsies, the extra follow-ups (in BI-RADS 3 cases) will presumably also cause a psychological impact [14].

Based on these findings, it appears that mammography in the setting of focal breast complaints must be regarded as a screening examination with a very low yield (1/494 = 0.002%) and a relatively high false positive rate (28/445 = 0.06%) in women with benign outcome with regard to the focal complaint, that compares poorly to national screening programs.

There are several limitations of our retrospective study. The reports of mammography and ultrasound were in practice sometimes combined and the ultrasound findings were interpreted at the time of already known outcome of the mammography. In some cases it wasn’t always “easy” to identify the symptomatic lesion (if there were more lesions) on mammography because there was no marker used which correlated to the complaint. Another limitation is the lack of subgroup analyses because of the small sample size. Furthermore, the follow-up period was not very long (only 24 months after inclusion).

Our findings suggest that in women with focal breast complaints it might be better to use mammography on indication than as standard initial evaluation technique. The decision whether mammography is required could be made after interpretation of the targeted ultrasound due to the high negative predictive value of this examination. This is in line with other studies [15–17]. Such an approach will prevent the performance of mammography in case of clearly benign outcomes according to the targeted ultrasound (BI-RADS 1 or 2), also improving the cost-effectiveness of the breast imaging clinic.

## Conclusion

Breast radiologists are well capable to distinguish “typical” benign lesions from malignant lesions using targeted ultrasound alone in women with focal breast complaints. The added value of mammography in patients with focal breast complaint is generally very low. Further, prospective evaluation of our findings is, however, required.

## Appendix

See Table 2.

**Table 2:** Different types of detected cancers and biopsied benign lesions

	Symptomatic lesions	Additional lesions
Total biopsied lesions	86	24
Biopsied benign	38	18
Normal breast tissue	2	–
Fibroadenoma	9	5
Phyllodes	2	–
Fibrosis/adenosis	11	3
Cyst (complex)/hyperplasia	5	6
Scar lesion	1	–
Hematoma	3	–
PASH	1	1
Other	4	3
Biopsied malignant	48	1
Intraductal papilloma with DCIS	1	–
DCIS	–	1
IDC (with DCIS)	40	–
ILC (with DCIS)	7	–
Other	–	–

## Abbreviations

BI-RADS: Breast imaging reporting and data system
DCIS: Ductal carcinoma in situ
MRI: Magnetic resonance imaging
PPV: Positive predicting value
